# AEGIS: A Semantic GAN and Evidential Learning Framework for Robust Adversarial Detection in Vision Sensors

**DOI:** 10.3390/s26154729

**Published:** 2026-07-25

**Authors:** Maher Boughdiri, Mounira Msahli, Albert Bifet

**Affiliations:** Laboratoire des Traitement et de Communication de l’Information, Département Informatique et Réseaux, Télécom Paris, Institut Polytechnique de Paris, 91120 Palaiseau, France; mounira.msahli@telecom-paris.fr (M.M.); albert.bifet@telecom-paris.fr (A.B.)

**Keywords:** adversarial defense, deep learning, GAN, evidential learning, Tiny ImageNet

## Abstract

Deep neural networks (DNNs) have shown outstanding performance in visual recognition tasks within vision sensor networks; however, they are still vulnerable to adversarial manipulations and imperceptible perturbations that can lead to erroneous predictions. To address that, this paper presents AEGIS, a semantic aware and uncertainty guided adversarial detection framework designed for robust image classification in vision sensors pipelines. At its core, a SemantiGAN module functions as a multi-class semantic discriminator, identifying and filtering visually inconsistent adversarial inputs before they propagate further in the pipeline. For inputs that pass this stage, a stochastic augmentation process generates test time variations, from which handcrafted instability metrics FlipScore, Prediction Inconsistency, Layerwise Cosine Similarity (early and mid layers), and Entropy are computed. These features are aggregated into a compact five dimensional vector and processed by an Evidential Deep Learning (EDL) classifier, which models output evidence using a Dirichlet distribution to yield both class predictions and calibrated uncertainty estimates. Evaluations on the Tiny ImageNet dataset across six categories clean, FGSM, PGD, patch-based, functional, and geometric attacks demonstrate the effectiveness of AEGIS. The proposed framework achieves an AUROC of 92.1%, an AUPRC of 90.2%, and an accuracy of 90.7%, outperforming conventional softmax-based detectors in terms of detection performance, robustness, interpretability, and uncertainty calibration.

## 1. Introduction

DNNs have become the cornerstone of modern computer vision systems and are increasingly deployed in safety-critical applications such as autonomous driving, facial recognition, medical imaging, and intelligent surveillance [1]. Despite their remarkable success, DNNs remain highly vulnerable to adversarial attacks, where carefully crafted perturbations can induce incorrect predictions while remaining nearly imperceptible to human observers [2,3]. Such vulnerabilities pose significant safety and security risks. For instance, a manipulated stop sign may be misclassified as a speed limit sign, potentially leading to hazardous decisions in autonomous navigation systems [4]. Furthermore, the transferability of adversarial examples enables successful black-box attacks without requiring direct access to the target model, thereby increasing the practical threat posed by adversarial manipulation [5].

Early adversarial attacks primarily focused on pixel-level perturbations constrained by $l_{p}$ norms [6]. Representative methods such as the Fast Gradient Sign Method (FGSM) and Projected Gradient Descent (PGD) generate subtle modifications that preserve the visual appearance of the original image while misleading the classifier [7]. More recently, adversarial research has evolved beyond low-level perturbations toward higher-level semantic manipulations [8,9]. These include adversarial patches, geometric transformations, spatial distortions, and functional attacks that alter contextual relationships while preserving human-recognizable content [10,11,12,13]. Unlike traditional perturbation-based attacks, semantic adversarial examples exploit weaknesses in contextual reasoning, shape understanding, and texture biases, making them considerably more challenging to detect and defend against [14,15].

To counter these threats, numerous defense mechanisms have been proposed, including input preprocessing, adversarial training, gradient masking, anomaly detection, and uncertainty estimation [16,17,18]. However, most existing approaches focus on a single aspect of adversarial behavior and often fail to generalize across heterogeneous attack categories. Pixel-level defenses are generally ineffective against patch-based or semantic attacks, while methods designed for $l_{p}$-bounded perturbations remain vulnerable to functional and geometric manipulations [10,14,19]. Moreover, many current defenses provide limited interpretability and uncertainty awareness, reducing their reliability in safety-critical environments where trustworthy decision-making is essential.

The limitations of existing defenses highlight three key requirements for robust adversarial detection. First, semantic consistency analysis is needed to identify attacks that manipulate contextual or structural information while preserving visual realism [20,21]. Second, instability profiling can reveal adversarial behavior by measuring how predictions and internal representations change under benign transformations [22]. Third, uncertainty-aware reasoning is necessary to prevent overconfident predictions and enable the safe rejection of suspicious inputs [23].

To address these gaps, the main contribution of this work is the proposal of AEGIS, a unified adversarial detection framework that combines semantic reasoning, instability analysis, and evidential uncertainty modeling. It consists of three complementary modules: (1) SemantiGAN, a GAN-based semantic discriminator that performs multi-class adversarial filtering; (2) LAFANet, an instability profiling module that extracts handcrafted features from test-time augmentations; and (3) Evidential Deep Learning, which provides calibrated classification and epistemic uncertainty estimation through a Dirichlet-based formulation. By jointly leveraging semantic consistency, prediction instability, and uncertainty awareness, AEGIS offers a comprehensive defense against both low-level perturbation attacks and high-level semantic manipulations.

The remainder of this paper is structured as follows. Section 2 surveys related work in adversarial detection and defenses approaches. Section 3 defines the adversarial threat model and security objectives. Section 4 presents our full methodology, including SemantiGAN, LAFANet features, and the EDL classifier. Section 5 details the experimental setup and evaluation metrics. Then, Section 6 discusses results, security implications, and ablation studies. Finally, we conclude with future directions.

## 2. Related Work

In this section, recent advances in adversarial attack detection and defense strategies in sensors networks are reviewed.

### 2.1. Adversarial Detection Methods

#### 2.1.1. Confidence and Statistical-Based Detection

Early adversarial detection methods relied on confidence-based indicators such as softmax probabilities and predictive entropy, assuming that adversarial perturbations reduce model confidence [17,18]. However, adaptive attacks can maintain high confidence while still causing misclassification, significantly limiting the effectiveness of these approaches [24]. To overcome this limitation, statistical methods such as Mahalanobis distance [25] and Local Intrinsic Dimensionality (LID) [26] were introduced to identify deviations in feature-space distributions between clean and adversarial samples. Although these methods achieve promising performance in controlled environments, their effectiveness often deteriorates on deeper architectures and complex high-dimensional datasets.

#### 2.1.2. Transformation and Instability-Based Detection

A growing body of work exploits the instability of adversarial examples under benign input transformations. These methods leverage Test-Time Augmentation (TTA) and stochastic perturbations to assess prediction consistency across multiple transformed views [27,28]. Approaches such as feature squeezing [29], prediction flipping analysis [30], and density-aware normalization [31,32] quantify instability through metrics including entropy, prediction variance, and label inconsistency. More recent studies investigate feature-space divergence, frequency-domain transformations, and stochastic ensemble strategies to improve robustness against adaptive attacks [33,34,35]. Despite their effectiveness, most transformation-based detectors rely on a limited set of instability measures and lack explicit semantic reasoning, reducing their ability to identify semantically aligned adversarial manipulations.

To address the limitations of purely statistical approaches, recent research has incorporated semantic information into adversarial detection. Methods based on attention alignment, scene coherence modeling, and semantic graph reasoning aim to identify violations of semantic consistency rather than low-level perturbation artifacts [36,37,38]. These approaches demonstrate improved robustness against structurally consistent attacks; however, they are often computationally expensive, highly dependent on dataset-specific semantics, and difficult to generalize across diverse attack families.

### 2.2. Adversarial Defense Mechanisms

#### 2.2.1. GAN-Based Defenses

Generative Adversarial Networks (GANs) have been widely adopted as defense mechanisms against adversarial attacks. A common strategy consists of projecting adversarial samples back onto the learned data manifold before classification. Defense-GAN [39] exemplifies this approach through iterative latent-space optimization, while anomaly-detection frameworks such as GANomaly [40], G-VAE [41], and RGAnomaly [42] leverage reconstruction errors and latent-space deviations to identify abnormal inputs. Although effective in capturing low-level perturbations, reconstruction-based approaches often suffer from high computational overhead, reconstruction artifacts, and reduced effectiveness against semantically coherent attacks such as adversarial patches.

More recently, researchers have explored GAN discriminators as direct adversarial detectors. AdvGAN [43] employs a discriminator to enforce perceptual realism during adversarial generation, while class-conditional GANs improve class-aware discrimination [44]. Advanced architectures such as GEUN [45] and FSD-GAN [46] integrate multiple discriminative modules to improve robustness against manipulated content. Nevertheless, existing discriminator-based approaches are generally designed for specific attack scenarios and rarely exploit semantic adversarial categories as an explicit detection objective.

#### 2.2.2. Uncertainty-Aware Learning for Robust Detection

Conventional neural networks rely on softmax outputs that are often poorly calibrated and prone to overconfidence, particularly when processing adversarial or out-of-distribution samples [47,48]. Bayesian approaches such as Monte Carlo Dropout [49] and Deep Ensembles [50] estimate predictive uncertainty by approximating posterior distributions over model parameters. Although effective, these methods typically require multiple forward passes and significant computational resources.

Evidential Deep Learning (EDL) [51] provides a computationally efficient alternative by modeling classifier outputs as evidence parameters of a Dirichlet distribution. This framework jointly estimates class probabilities and epistemic uncertainty within a single forward pass. Recent studies have demonstrated that EDL improves robustness in adversarial and out-of-distribution detection tasks while providing more reliable uncertainty estimates than conventional confidence-based measures [52,53,54].

### 2.3. Research Gaps

Despite significant progress, existing approaches typically address only isolated aspects of adversarial robustness. Statistical detectors focus on distributional anomalies, transformation-based methods exploit prediction instability, semantic-aware approaches analyze contextual consistency, GAN-based defenses perform purification or specialized discrimination, and uncertainty-aware models provide calibrated confidence estimates. To the best of our knowledge, no prior framework jointly integrates semantic adversarial discrimination, transformation-driven instability profiling, and evidential uncertainty calibration within a unified and computationally efficient architecture. AEGIS addresses this gap by combining SemantiGAN, LAFANet, and Evidential Deep Learning into a single end-to-end framework capable of recognizing six adversarial categories while providing calibrated uncertainty estimates for reliable decision-making.

## 3. Threat Model

### 3.1. Adversary Capabilities and Attack Modalities

**Figure 1:**
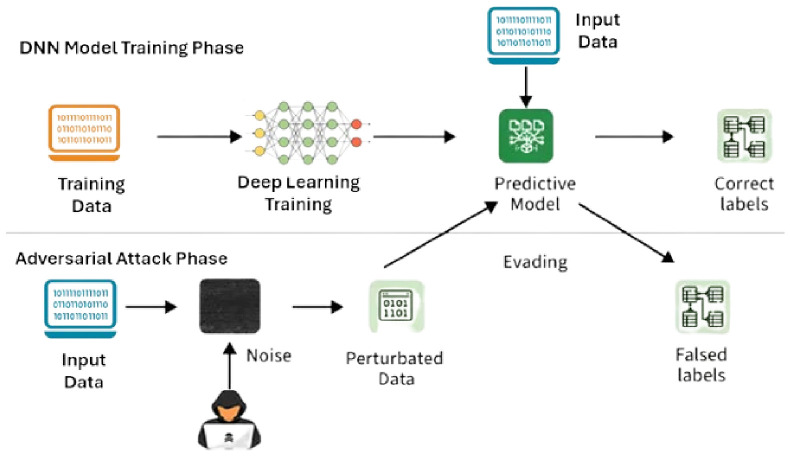
Adversarial Deep Learning Attacks.

Adversarial attacks refer to intentional manipulations of input data designed to mislead a machine learning model into making incorrect predictions while preserving the visual appearance of the input, as illustrated in Figure 1. These attacks introduce small, often imperceptible perturbations that exploit weaknesses in the model’s decision process. As a result, adversarial perturbations pose a persistent security risk to deep learning-based vision pipelines, particularly in real-time deployments where high-confidence misclassifications can trigger safety-critical failures [55,56]. Such perturbations are crafted to alter the model’s output without changing the human-perceived semantics of the image, thereby undermining the trustworthiness of DNN-enabled decision systems.

Adversarial attacks can be categorized based on the attacker’s level of knowledge into three settings: white-box [57], black-box [58], and gray-box [59]. In this work, a white-box adversary is considered, assuming complete access to the target model’s architecture, parameters, and gradients. This level of access enables the use of loss-driven backpropagation to construct adversarial perturbations that maximize the probability of misclassifications. Such a setting represents a strong, worst-case scenario and is widely adopted to evaluate the upper bounds of model robustness.

The adversary’s objective is to generate perturbed inputs that induce incorrect or targeted predictions while preserving perceptual similarity, ensuring that the modifications remain imperceptible to human observers. These perturbations are typically constrained under a bounded norm (e.g., $l_{p}$), or designed to maintain semantic plausibility in more advanced attack scenarios. Under this threat model, the following classes of adversarial attacks are considered:

- $l_{p}$-bounded gradient-based attacks: Methods such as Fast Gradient Sign Method (FGSM) and Projected Gradient Descent (PGD) [60,61] exploit gradient information to compute adversarial perturbations that maximize the loss function with respect to the input. These perturbations are constrained within a predefined norm ball (e.g., $l_{\infty }$, $l_{2}$), ensuring imperceptibility while effectively pushing samples across decision boundaries. PGD, as an iterative extension of FGSM, is widely regarded as a first-order worst-case adversary due to its stronger optimization capability.
- Patch-based attacks: These attacks introduce localized, high-impact perturbations confined to a small region of the input image [10,62]. Unlike global perturbations, adversarial patches are optimized to dominate salient features and attention mechanisms, often overriding the original content. Their spatial locality and robustness make them particularly effective in both digital and physical settings.
- Functional (semantic) attacks: Rather than relying on low-level noise, these attacks manipulate high-level attributes such as object texture, material properties, or contextual relationships [63]. The resulting samples remain visually plausible to humans but induce semantic inconsistencies that mislead the model, revealing its reliance on non-robust or spurious correlations.
- Geometric transformation attacks: These attacks apply spatial transformations, including rotations, translations, scaling, and non-linear warpings [64]. Such transformations exploit the limited invariance of convolutional neural networks, leading to significant changes in internal feature representations despite minimal perceptual distortion.
- Transferable adversarial examples: Although commonly associated with black-box settings, transferability can also be studied under white-box conditions by crafting perturbations that generalize across models. These adversarial examples exploit shared decision boundaries and feature representations, highlighting systemic vulnerabilities in deep learning models [65].
- Hybrid/zero-day attacks: These attacks combine multiple perturbation strategies (e.g., gradient-based noise with geometric distortion or semantic manipulation) to produce more complex and evasive adversarial examples. Such compositions often fall outside the training distribution and challenge detection mechanisms, representing realistic and previously unseen (zero-day) threat scenarios [24].

### 3.2. Security Landscape and Design Constraints

Beyond conventional adversarial attacks, the deployment of vision-based AI systems in real-world environments introduces additional security and operational challenges that must be considered during the design of adversarial defense mechanisms. A  critical challenge is adversarially induced overconfidence, where adversarial inputs not only trigger incorrect predictions but also produce artificially high confidence scores, concealing the model’s uncertainty and potentially misleading downstream decision-making processes [66]. Such behavior is especially problematic in safety-critical applications, where confidence estimates are often used to trigger automated actions or risk mitigation procedures.

An effective adversarial defense must simultaneously satisfy three core requirements: robustness against a wide range of adversarial manipulations, compliance with real-time inference constraints, and the ability to provide transparent and interpretable decision-making. AEGIS is designed to address these requirements through a modular architecture comprising three complementary components. First, SemantiGAN enables early rejection of semantically inconsistent or out-of-distribution inputs by modeling class-level semantic coherence. Second, LAFANet leverages instability metrics derived from lightweight test-time augmentations to expose hidden sensitivity to adversarial perturbations, including gradient-based and geometric distortions. Third, EDL provides calibrated predictions through principled uncertainty estimation, enabling reliable rejection of ambiguous or adversarially influenced inputs. Each module in AEGIS is explicitly aligned with a distinct adversarial capability: SemantiGAN targets semantic and patch-based attacks by filtering visually inconsistent inputs, LAFANet captures vulnerability to gradient and geometric perturbations through prediction instability, and EDL addresses adversarially induced overconfidence by quantifying epistemic uncertainty. This unified design mitigates a broad spectrum of modeled threats; however, residual risks remain in more complex settings such as physical-world robustness and adaptive zero-day attacks, which are further discussed in Section 6.

## 4. AEGIS Framework

**Figure 2:**
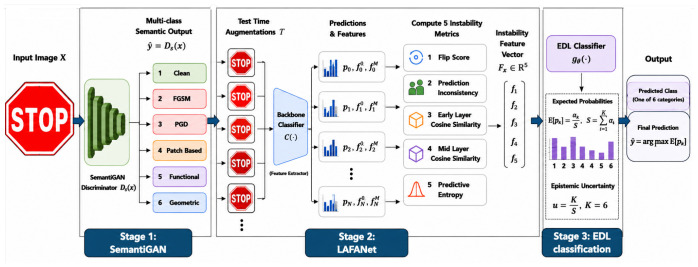
Overview of AEGIS adversarial detection framework.

AEGIS is a modular adversarial detection and classification framework that integrates three complementary components: (i) SemantiGAN a multi-class semantic discriminator based on a generative adversarial network, (ii) LAFANet an instability profiling module extracting handcrafted features from test time augmentations, and (iii) Evidential Deep Learning a calibrated classifier that models epistemic uncertainty. The overall pipeline, illustrated in Figure 2, processes every input image through all three stages, ensuring that both clean and adversarial inputs are consistently analyzed for final classification across six categories. In the first stage, SemantiGAN performs multi-class semantic discrimination by recognizing different categories of adversarial perturbations and providing a coarse semantic characterization of the input. However, semantic discrimination alone is insufficient to reliably determine whether an image is truly adversarial, as different attack types may exhibit similar semantic characteristics. To address this limitation, the second stage employs LAFANet, which analyzes prediction instability under stochastic, label-preserving augmentations and extracts a set of handcrafted instability features. Finally, these features are provided to the EDL classifier, which produces the final adversarial classification while quantifying epistemic uncertainty. This uncertainty-aware decision process improves the reliability, robustness, and interpretability of the framework, particularly when confronted with ambiguous, adversarial, or out-of-distribution inputs.

### 4.1. Framework Stages

The AEGIS framework is composed of three stages: (1) SemantiGAN, a multi-class semantic discriminator; (2) Augmentation-Driven Instability Profiling (LAFANet); and (3) Evidential Deep Learning (EDL) Classification.

#### 4.1.1. Stage 1: SemantiGAN—Multi-Class Semantic Discriminator

The first stage employs a semantic GAN discriminator $D_{s}\left(\cdot \right)$ trained to classify an input image *x* into one of K=6 semantic categories:

(1) $$\hat{y}=D_{s}\left(x\right)=\mathrm{softmax}\left(f_{\theta }\left(x\right)\right)\in R^{6},$$

where $f_{\theta }$ denotes the discriminator’s feature extractor. The six categories include: clean, FGSM, PGD, patch based, functional, and geometric adversarial examples.

Unlike traditional GAN discriminators optimized for binary real/fake detection, SemantiGAN is directly trained for multi-class adversarial recognition, enabling it to learn high level semantic differences between diverse attack types and clean inputs.

The output from SemantiGAN serves as a coarse semantic cue that complements the downstream instability analysis.

#### 4.1.2. Stage 2: Augmentation Driven Instability Profiling (LAFANet)

Following semantic classification, the image is passed to LAFANet, which evaluates prediction stability under stochastic, label-preserving transformations.

These transformations are defined by a set of lightweight operators $T=\{T_{1},T_{2},\ldots ,T_{M}\}$. T denotes a predefined set of stochastic, label-preserving transformation operators from which transformations are sampled to generate augmented views of the input image. These transformations include horizontal flips, affine warps, brightness adjustments, motion blur, cutout, and JPEG compression.

Given an input image *x*, *N* stochastic augmentations are generated as:

(2) $${\left\{x_{i}\right\}}_{i=1}^{N}=T\left(x\right)$$

Each image (original and augmented) is processed by a backbone classifier C(·) producing class probability distributions:

(3) $$p_{0}=C\left(x\right),\;p_{i}=C\left(x_{i}\right)=\left[p_{i,1},p_{i,2},\ldots ,p_{i,K}\right]\in R^{K},$$

where $p_{i,j}$ denotes the predicted probability of class *j* for the *i*-th augmented image $x_{i}$, with $\sum_{j=1}^{K}p_{i,j}=1$.

The corresponding predicted labels are:

(4) $${\hat{y}}_{0}=\arg\max p_{0},\;{\hat{y}}_{i}=\arg\max p_{i,j}$$

From these outputs, five instability metrics are computed. These metrics are defined as follows.

Flip Score: Measures how often the predicted class changes under a stochastic augmentation $T_{i}$. Mainly, its a binary indicator of label change under horizontal flip [67].

(5) $$FlipScore\left(x\right)=\frac{1}{N}\sum_{i=1}^{N}1\left[\arg\max p_{i,j}\neq \arg\max p_{0}\right]$$

Prediction Inconsistency: Measures the dispersion of predicted labels across augmentations. It is the fraction of augmented views disagreeing with the majority prediction [68].

(6) $$Inconsistency\left(x\right)=1-\frac{\max_{c}\sum_{i=1}^{N}1\left[{\hat{y}}_{i}=c\right]}{N}$$

where 1[·] denotes the indicator function, equal to 1 when the specified condition is satisfied and 0 otherwise.

Feature Stability: Measures consistency of learned representations under augmentations: Cosine similarity between early-layer features of each view and the mean early-layer feature [69]; and the Cosine similarity between mid-layer features of each view and the mean mid-layer feature [70].

(7) $$EarlyCosSim\left(x\right)=\cos\left(f^{E}\left(x\right),{\bar{f}}^{E}\left(x\right)\right),\;MidCosSim\left(x\right)=\cos\left(f^{M}\left(x\right),{\bar{f}}^{M}\left(x\right)\right)$$

where

$${\bar{f}}^{E}=\frac{1}{N}\sum_{i=1}^{N}f^{E}\left(x_{i}\right),\;{\bar{f}}^{M}=\frac{1}{N}\sum_{i=1}^{N}f^{M}\left(x_{i}\right)$$

${\bar{f}}^{E}$ and ${\bar{f}}^{M}$ denote mean early- and mid-layer features over augmentations.

Predictive Entropy: Measures uncertainty in the predicted class distribution across augmentations [71].

(8) $$Entropy\left(x\right)=\frac{1}{N}\sum_{i=1}^{N}\left(-\sum_{j=1}^{K}p_{ij}\log p_{ij}\right)$$

where $p_{ij}$ denotes the predicted probability of class *j* for augmentation $x_{i}$.

The resulting 5D instability feature vector is:

(9) $$F_{x}=\left[FlipScore,\;Inconsistency,\;EarlyCosSim,\;MidCosSim,\;Entropy\right]$$

#### 4.1.3. Stage 3: Evidential Deep Learning (EDL) Classification

The final stage of AEGIS performs uncertainty-aware adversarial classification using an Evidential Deep Learning (EDL) classifier [51]. Unlike conventional softmax classifiers that produce deterministic confidence scores, EDL models predictive uncertainty by interpreting network outputs as evidence supporting different hypotheses. This enables the framework to jointly estimate both class probabilities and the confidence associated with those predictions.

In this stage, the 5D feature vector $F_{x}$ is provided as input to the EDL classifier, which produces non negative evidence $e\in R_{\geq 0}^{K}$. The corresponding Dirichlet parameters are:

(10) α=e+1,

The expected probability for each class is computed as:

(11) $$E\left[p_{i}\right]=\frac{\alpha_{i}}{S},\;S=\sum_{j=1}^{K}\alpha_{j},$$

where *S* denotes the total accumulated evidence.

Epistemic uncertainty is computed as:

(12) $$u=\frac{K}{S}.$$

Here, epistemic uncertainty refers to uncertainty arising from insufficient model knowledge, in contrast to aleatoric uncertainty, which originates from inherent noise in the observations. In adversarial detection, epistemic uncertainty is particularly important because adversarial or out-of-distribution inputs often lie outside the learned training distribution, leading to reduced evidence accumulation and consequently higher uncertainty scores.

The EDL classifier provides the final six-class prediction along with an uncertainty score. High uncertainty values can be flagged for human review or risk-aware decision-making in safety-critical deployments.

The EDL classifier transforms the raw evidence into a Dirichlet distribution that represents both the most likely class and how confident the model is about that choice. A high evidence value indicates strong confidence, while low evidence reflects greater uncertainty. This provides a principled way to reject inputs that may be adversarial or ambiguous.

In this stage, the 5-dimensional instability feature vector $F_{x}$, extracted by LAFANet, is provided as input to the EDL classifier:

(13) $$e=g_{\phi }\left(F_{x}\right)\in R_{\geq 0}^{K},$$

where

- $g_{\phi }\left(\cdot \right)$ denotes the evidential classifier,
- K=6 corresponds to the adversarial categories,
- and each component $e_{i}$ represents the amount of evidence supporting class *i*.

The evidence vector is then transformed into Dirichlet distribution parameters:

(14) $$\alpha_{i}=e_{i}+1,$$

The resulting Dirichlet distribution:

(15) Dir(p∣α),

provides a probabilistic representation over class predictions, enabling the model to jointly capture belief strength and predictive uncertainty.

Based on the resulting Dirichlet parameters, the expected class probabilities and epistemic uncertainty are computed according to Equations (11) and (12), respectively. The expected probability $E[p_{i}]$ represents the predictive belief assigned to class *i* and is used to determine the most likely adversarial category, whereas *u* quantifies the epistemic uncertainty associated with the prediction. These two quantities provide complementary information: the expected probabilities characterize what the model predicts, while the uncertainty score indicates how certain the model is about that prediction. Higher values of *u* correspond to lower accumulated evidence and therefore greater uncertainty.

The final adversarial prediction is obtained as:

(16) $$\hat{y}=\arg\max_{i}E\left[p_{i}\right].$$

The EDL classifier therefore transforms the extracted instability features into a Dirichlet-based evidential representation that simultaneously models the most likely adversarial category and the confidence associated with that prediction. High evidence values indicate strong support for a specific class, whereas low evidence reflects ambiguity or insufficient model confidence. This provides a principled mechanism for identifying unreliable predictions and rejecting potentially adversarial or ambiguous inputs. Consequently, samples associated with high uncertainty may be flagged for human inspection or risk-aware decision-making in safety-critical deployments such as autonomous driving, intelligent surveillance, industrial robotics, and medical imaging systems.

### 4.2. Design Rationale and Architectural Motivation

The AEGIS framework is designed according to a progressive adversarial analysis strategy that combines semantic reasoning, instability profiling, and uncertainty-aware decision making. Although SemantiGAN, LAFANet, and Evidential Deep Learning (EDL) can each operate independently as adversarial detection mechanisms, their sequential integration enables a more comprehensive characterization of adversarial behavior across semantic, statistical, and probabilistic dimensions. As presented in Algorithm 1, the first stage employs SemantiGAN as a semantic discriminator that analyzes the high-level structure and contextual consistency of the input image. This stage serves as an initial filtering mechanism by identifying semantic discrepancies associated with adversarial manipulations. Such a design is particularly effective against patch-based, functional, and geometric attacks that alter semantic representations while preserving visual plausibility. Performing semantic analysis first is computationally efficient and provides coarse adversarial priors that guide subsequent stages. The second stage utilizes LAFANet to evaluate the stability of model predictions under a set of lightweight stochastic augmentations. While semantically plausible adversarial examples may evade the first stage, they frequently exhibit unstable behavior when subjected to benign transformations. LAFANet captures this phenomenon through handcrafted instability metrics that quantify prediction consistency, feature-space coherence, and uncertainty variations. Compared with computationally intensive approaches such as Mahalanobis distance estimation, Local Intrinsic Dimensionality (LID), or ensemble-based detectors, LAFANet offers a lightweight and architecture-agnostic mechanism for exposing adversarial sensitivity. The final stage employs an EDL classifier to transform the extracted instability representation into class predictions accompanied by calibrated uncertainty estimates.

Unlike conventional softmax classifiers, which often produce overconfident predictions for adversarial or out-of-distribution samples, EDL explicitly models evidence and epistemic uncertainty through a Dirichlet distribution. Consequently, the framework can distinguish between confident adversarial classifications and ambiguous inputs for which insufficient evidence exists.
**Algorithm 1** AEGIS: End to End Six Class Adversarial Classification**Input**: Image *x* **Output**: (Predicted class $c^{*}$, Uncertainty *u*) **1:** ${\hat{y}}_{\mathrm{sem}}\leftarrow D_{s}\left(x\right)$ *SemantiGAN coarse prediction* **2:** Apply *N* stochastic augmentations to *x*;
**3: For** each $x_{i}$, extract ${\hat{y}}_{i}$, $f^{E}\left(x_{i}\right)$, $f^{M}\left(x_{i}\right)$
**4:** Compute FlipScore, Inconsistency, EarlyCosSim, MidCosSim, Entropy; form $F_{x}$.
**5:** $\left(e,u\right)\leftarrow \mathrm{EDL}\left(F_{x}\right)$; compute α and $E[p_{i}]$.
**6:** $c^{*}\leftarrow \arg\max E\left[p_{i}\right]$;
**return** $\left(c^{*},u\right)$.


## 5. Experimental Setup

### 5.1. System Configuration

All experiments were conducted on a high-performance workstation running Windows 11, equipped with an Intel64 processor (Family 6, Model 183) featuring 20 physical cores and 28 logical threads, 32 GB of RAM, and an NVIDIA RTX 2000 Ada Generation Laptop GPU with CUDA 12.8 support. The AEGIS framework was implemented in Python 3.10 using PyTorch 2.7.0+cu128 and TorchVision 0.16.0 for model development and training. Data augmentation and image transformation pipelines were implemented using Albumentations 1.3, while NumPy was employed for instability feature extraction and vector construction. All stages of the framework, including SemantiGAN training, LAFANet instability profiling, and Evidential Deep Learning (EDL) classification, were executed locally using single-GPU acceleration. This experimental configuration provided sufficient computational resources for adversarial example generation, large-scale augmentation processing, semantic adversarial classification, instability profiling, and uncertainty-aware evaluation across all six semantic classes.

### 5.2. Dataset

To evaluate the effectiveness of the AEGIS framework, we use the Tiny ImageNet-200 dataset, a widely adopted benchmark for image classification and adversarial learning. Tiny ImageNet consists of 200 object categories selected from the larger ImageNet hierarchy, with each class containing 500 training images, 50 validation images, and 50 test images. All images are resized to a resolution of 64×64 pixels, preserving the semantic diversity of ImageNet while remaining computationally efficient. For our experiments, we retain the original class distribution while repurposing the validation and test splits to include adversarial variants. The training split is primarily composed of clean images and is used to train the backbone classifier, LAFANet, and the EDL classifier, as illustrated in Table 1. However, the SemantiGAN module requires supervision across all six semantic categories (clean, FGSM, PGD, patch-based, functional, and geometric attacks). Therefore, adversarial examples were generated from the training split and used exclusively to train SemantiGAN. In contrast, both LAFANet and the EDL classifier were trained solely on clean images. During training, LAFANet learns the characteristic stability patterns of benign samples under stochastic augmentations, while the EDL module learns to map the resulting instability representations to calibrated evidential outputs. This design ensures a clear separation between semantic adversarial recognition and instability-based uncertainty modeling. Specifically, SemantiGAN is trained to recognize adversarial attack categories, whereas LAFANet and EDL operate under a clean-data training regime and detect adversarial behavior through deviations from normal prediction and feature stability patterns. Further, to prevent cross-contamination between the framework stages, the images used to generate adversarial examples for SemantiGAN training are different from those used to generate adversarial examples for evaluating LAFANet and the EDL classifier. Specifically, adversarial training samples are synthesized exclusively from the training split, whereas all evaluation adversarial samples are generated exclusively from the test split. Therefore, no image or adversarial variant appears in both training and testing, ensuring a strict separation between all stages of the proposed framework.

The validation set is used for hyperparameter tuning and early stopping. The test set is augmented with multiple adversarial versions of the original images, enabling robust evaluation of multi-class detection performance across clean and perturbed data. To ensure a balanced evaluation, we construct a six-class test set where each original image is associated with five corresponding adversarial variants generated using different attack methods. The resulting test set includes clean samples and adversarial examples from FGSM, PGD, patch-based, functional, and geometric attacks. The adversarial portion of the test set contains 50,000 samples, evenly partitioned with 10,000 samples per attack type. Adversarial examples for evaluation were generated exclusively from the test set to avoid train–test contamination, whereas adversarial examples for training SemantiGAN were synthesized from the training split.

### 5.3. Training Strategy

The complete training configuration of the proposed AEGIS framework is described as follows. SemantiGAN adopts a multi-path convolutional architecture comprising both a generator and a discriminator. The discriminator is trained as a six-class semantic classifier using the categorical cross-entropy loss and the Adam optimizer with a learning rate of $1\;\times \;10^{-4}$, $\beta_{1}=0.5$, and $\beta_{2}=0.999$. Training is performed for 100 epochs with a batch size of 128. To improve generalization and reduce overfitting, a dropout rate of 0.3 and a weight decay of $1\;\times \;10^{-5}$ are employed. LAFANet is trained exclusively on clean images to learn stable instability representations from multiple semantic-preserving augmentations. The network is optimized using the standard cross-entropy classification loss, enabling it to characterize the normal prediction stability of benign samples without exposure to adversarial examples during training. The EDL classifier is implemented as a three-layer multilayer perceptron (MLP) with hidden dimensions of 128, 64, and 32 neurons, using ReLU activation functions in the hidden layers and a Softplus activation at the output to generate non-negative evidence values. The network is trained using the Adam optimizer with a learning rate of $5\times 10^{-4}$, a batch size of 64, and 80 epochs. The optimization objective is the standard evidential loss, which combines a mean squared error (MSE) term with a Kullback–Leibler (KL) divergence regularization term to simultaneously improve classification accuracy and produce well-calibrated uncertainty estimates. The proposed AEGIS framework is trained using a stage-wise training strategy rather than end-to-end global optimization function. Specifically, the backbone classifier is first trained on clean images, followed by SemantiGAN using clean and adversarial samples generated exclusively from the training split. After convergence, the parameters of SemantiGAN are frozen, and LAFANet is trained exclusively on clean images. Finally, the EDL classifier is trained using the instability feature vectors produced by the frozen LAFANet. This sequential strategy allows each module to optimize its task-specific objective independently while preventing gradient interference among heterogeneous learning objectives. In the Hyperparameter tuning approach, the final hyperparameters were selected according to the best validation accuracy and calibration performance. Learning rates, batch sizes, dropout rate, and weight decay were optimized according to the validation accuracy and ECE error. Early stopping based on validation loss was employed to prevent overfitting.

### 5.4. Augmentation Protocol and Instability Metrics

Stage 2 of AEGIS employs a lightweight, label-preserving augmentation pipeline to induce controlled variability in test-time predictions. The augmentation set, implemented using the Albumentations library, includes spatial transformations such as horizontal flips and random affine transformations, photometric perturbations including brightness jitter and motion blur, content masking via cutout with random mask size and position, and compression noise through JPEG artifact injection.

Each input image generates N=10 augmented views ${\left\{x_{i}\right\}}_{i=1}^{N}$, which are processed through a fixed backbone network to obtain predicted labels $\left\{{\hat{y}}_{i}\right\}$ along with early- and mid-level representations $\left\{f^{E}\left(x_{i}\right),f^{M}\left(x_{i}\right)\right\}$.

From these outputs, the five instability metrics are computed to form the LAFANet feature vector $F_{x}$, capturing complementary aspects of prediction variability, feature-space consistency, and model uncertainty. These handcrafted features quantify semantic and representational instability induced by adversarial perturbations while maintaining low computational overhead, making the approach both efficient and effective for robustness analysis.

Although AEGIS models heterogeneous adversarial perturbations through instability-aware representations extracted from semantic-preserving augmentations, alternative feature modeling strategies could also be explored. Recent advances in hyperspectral image restoration have demonstrated the effectiveness of sparse and low-rank priors for separating heterogeneous disturbance components [72], spectral learning preference for robust representation learning under complex noise distributions [73], and hybrid state-space and attention mechanisms for capturing both local and global perturbation patterns [74]. While these approaches were originally developed for hyperspectral image denoising, their underlying principles may provide valuable inspiration for future extensions of AEGIS. In particular, integrating structured priors, spectral-aware representation learning, or attention-guided feature extraction into the instability analysis stage could further improve the characterization of heterogeneous adversarial perturbations while preserving semantic consistency.

### 5.5. Evaluation Metrics and Baselines

We employ a diverse set of quantitative metrics to comprehensively evaluate detection accuracy, robustness, and trust calibration. The primary classification metric is overall accuracy across the six classes, which includes clean and all five adversarial types. In addition, we report macro averaged precision, recall, and F1 score to account for potential class imbalance and to capture fine grained detection fidelity. To assess binary detection capability distinguishing clean from adversarial we compute the Area Under the Receiver Operating Characteristic curve (AUROC), which provides a threshold independent measure of separability. We also analyze per class AUROC to determine the model’s sensitivity to specific attack types.

For uncertainty evaluation, we report the average epistemic uncertainty score produced by the evidential deep learning model for clean and adversarial samples. We further compute the Expected Calibration Error (ECE) to quantify the alignment between predicted confidence and true likelihood [75]. The ECE was defined as,

(17) $$\mathrm{ECE}=\sum_{m=1}^{M}\frac{|B_{m}|}{N}\left|\mathrm{acc}\left(B_{m}\right)-\mathrm{conf}\left(B_{m}\right)\right|,$$

where $B_{m}$ denotes the samples assigned to the *m*-th confidence bin, $\mathrm{acc}(B_{m})$ and $\mathrm{conf}(B_{m})$ represent the corresponding empirical accuracy and average confidence, respectively, and *N* is the total number of test samples. In our experiments, ECE is computed using the standard weighted formulation with M=15 equally spaced confidence bins [75]. No post hoc calibration technique, such as temperature scaling, is applied to either AEGIS or the softmax baseline. Consequently, all reported ECE values reflect the intrinsic calibration quality of each method.

## 6. Results Analysis and Discussions

This section presents a comprehensive evaluation of the AEGIS framework under diverse adversarial conditions. Performance is assessed using standard classification and detection metrics, including AUROC, AUPRC, F1 score, and accuracy, together with analyses of robustness, uncertainty calibration, and generalization. The objective is to evaluate not only the detection capability of AEGIS but also its ability to maintain reliable performance across heterogeneous adversarial threat models.

### 6.1. Quantitative Detection Performance

Table 2 summarizes the detection performance of AEGIS across five representative adversarial attack categories. Overall, the framework achieves an average AUROC of 91.8%, an average AUPRC of 89.98%, an F1 score of 89.32%, and an overall classification accuracy of 90.12%, demonstrating strong discrimination between clean and adversarial samples across multiple attack families.

Among the evaluated attacks, FGSM exhibits the highest detection performance, achieving an AUROC of 96.1% and an accuracy of 95.3%. This result is expected, as single-step gradient perturbations often introduce characteristic instability patterns that are effectively captured by the LAFANet features. PGD attacks remain highly detectable, achieving an AUROC of 94.2%, confirming that the proposed instability profiling mechanism remains effective against stronger iterative gradient-based attacks.

More challenging attack classes include patch-based, functional, and geometric perturbations. These attacks preserve much of the original image structure while manipulating semantic content or spatial relationships, making them inherently more difficult to detect. Nevertheless, AEGIS maintains AUROC values above 88% across all three categories, demonstrating the effectiveness of the combined semantic-discrimination and instability-analysis strategy. In particular, the performance on geometric attacks (88.3% AUROC) highlights the contribution of SemantiGAN in capturing semantic inconsistencies that may not be evident through prediction confidence alone.

### 6.2. Cross-Dataset Generalization

To evaluate the scalability and transferability of AEGIS beyond Tiny ImageNet, we conducted preliminary experiments on ImageNet-128. As shown in Table 3, the framework maintains strong performance despite the increased image resolution and dataset complexity. Detection accuracy decreases only marginally from 90.1% to 88.3%, while AUROC remains high at 89.7%. Furthermore, the Expected Calibration Error (ECE) increases only slightly from 1.6% to 2.2%, indicating that the EDL module preserves well-calibrated uncertainty estimates under more challenging conditions.

These results suggest that the proposed combination of semantic discrimination, instability profiling, and evidential uncertainty modeling generalizes effectively across datasets and resolutions, supporting its applicability to real-world computer vision systems operating in dynamic threat environments.

### 6.3. Discriminator Output and Semantic Filtering

As shown in Table 4, the SemantiGAN discriminator achieves high precision and recall in identifying adversarial samples while maintaining a low false rejection rate on clean inputs. By acting as an initial semantic filtering stage, it effectively reduces the burden on downstream modules and improves overall decision reliability. Its integration into the AEGIS pipeline increases downstream classification accuracy by more than 4.5%, highlighting its effectiveness as a semantic gatekeeper for adversarial robustness.

### 6.4. Uncertainty and Out-of-Distribution Detection

The Evidential Deep Learning (EDL) classifier within the AEGIS pipeline is evaluated for its ability to quantify uncertainty and produce calibrated predictions under adversarial perturbations. Unlike conventional softmax-based classifiers that tend to produce overconfident outputs, the proposed model estimates a Dirichlet distribution over class probabilities, enabling the detection of ambiguous or malicious inputs through reduced evidence accumulation and increased predictive entropy.

To evaluate this behavior, we report standard calibration and uncertainty metrics, including the Brier score, which measures the mean squared error between predicted confidence and ground-truth labels, and the Expected Calibration Error (ECE), which quantifies the misalignment between predicted confidence and empirical accuracy. In addition, predictive entropy is used as an indicator of uncertainty, where higher values correspond to less confident predictions. These metrics are computed separately for clean samples and each adversarial category, including FGSM, PGD, patch-based, geometric, and functional attacks.

As shown in Table 5, the classifier exhibits low uncertainty and strong calibration on clean data, while adversarial inputs consistently induce higher entropy and increased calibration error. These results demonstrate the effectiveness of the evidential formulation in distinguishing between epistemic uncertainty caused by adversarial perturbations and normal predictive variability.

### 6.5. Visualizations

**Figure 3:**
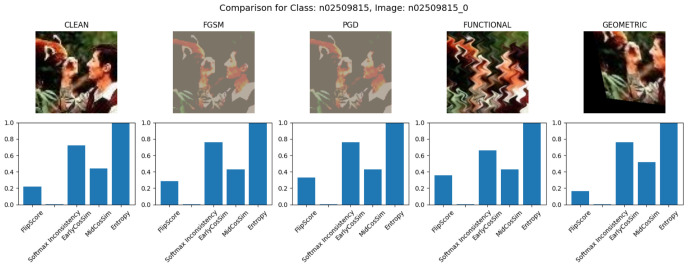
Qualitative comparison of instability metrics for clean and adversarial samples (example 1).

**Figure 4:**
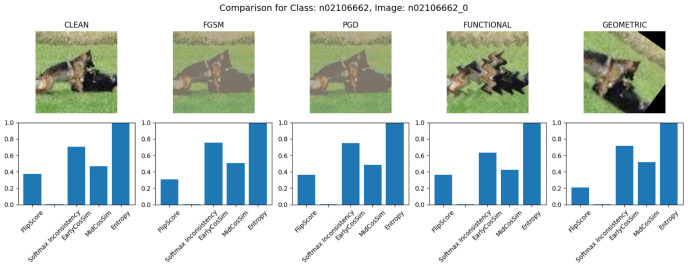
Qualitative comparison of instability metrics for clean and adversarial samples (example 2).

**Figure 5:**
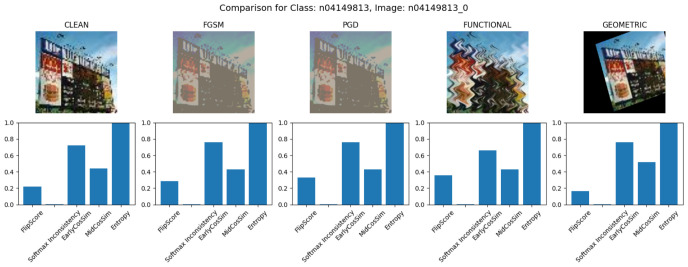
Qualitative comparison of instability metrics for clean and adversarial samples (example 3).

To provide deeper insights into how different adversarial perturbations influence model interpretability, we present qualitative visualizations of instability metrics across representative examples from the Tiny ImageNet dataset (Figure 3, Figure 4 and Figure 5). For each input image, we show the clean version alongside its adversarial counterparts generated using FGSM, PGD, functional, and geometric attacks. Beneath each image, bar charts illustrate the five handcrafted metrics computed by LAFANet: FlipScore, Inconsistency, EarlyCosSim, MidCosSim, and Entropy. These visualizations highlight how adversarial perturbations systematically increase prediction instability, reduce feature consistency, and amplify uncertainty compared to clean inputs.

### 6.6. Interpretability and Real-World Scenario

In AEGIS, interpretability is inherently provided through the five instability features computed by LAFANet, which are directly integrated into the decision-making pipeline. As illustrated in Figure 3, Figure 4 and Figure 5, small benign transformations cause adversarial inputs to exhibit frequent label changes, feature misalignment, and increased entropy, while clean inputs remain stable under the same perturbations. These effects are consistently captured by FlipScore, Inconsistency, cosine similarity measures, and predictive entropy. For each sample, these indicators provide a concise and quantitative rationale, showing that adversarial examples systematically exhibit higher instability and uncertainty than clean samples. Because these same features are used both for classification and uncertainty-based rejection, the resulting explanations are inherently transparent and easily auditable.

A representative deployment scenario involves a low-speed autonomous shuttle operating in urban environments, where traffic signs may be manipulated using adversarial stickers or patch-based perturbations. In such settings, AEGIS first filters semantically inconsistent frames using SemantiGAN, then computes instability features and an evidential uncertainty score *u*. If $u>\tau_{u}$, the system rejects the input and activates a safe fallback strategy (e.g., slowing down or stopping) while logging diagnostic evidence such as increased inconsistency, reduced cosine similarity, and elevated entropy. Otherwise, the system proceeds with the predicted class. This design is well suited for real-time, resource-constrained edge deployment scenarios.

While adversarial examples may appear visually emphasized in the presented figures for interpretability purposes, in practice such perturbations are often imperceptible to human observers and can bypass common preprocessing techniques such as JPEG compression or smoothing. Therefore, dedicated detection pipelines such as AEGIS remain necessary to ensure robustness against modern adversarial threats.

### 6.7. Security-Oriented Evaluations

In safety-critical applications, the robustness of adversarial detection systems must be evaluated not only against known attack distributions but also against previously unseen and transferable adversarial threats. Accordingly, this subsection presents a comprehensive security assessment of AEGIS, including its generalization to zero-day adversarial examples, resistance to cross-model transfer attacks, and effectiveness in mitigating the threat scenarios defined in the proposed threat model.

#### 6.7.1. Zero-Day Generalization

To evaluate the ability of AEGIS to detect previously unseen adversarial strategies, we simulate zero-day threats by generating hybrid perturbations that were not included during training. Specifically, we construct composite attacks combining PGD with geometric transformations such as random rotations and scaling. These hybrid adversarial samples are explicitly excluded from the training of both the SemantiGAN discriminator and the LAFANet module, ensuring a strict evaluation of out-of-distribution robustness. Detection is performed using the evidential classifier.

As shown in Table 6, AEGIS maintains strong detection performance under zero-day conditions, achieving an AUROC of 86.5% while exhibiting increased entropy for novel perturbations. This behavior confirms that both the semantic discriminator and evidential classifier retain meaningful generalization capability even when attack distributions deviate from training conditions.

Previous evaluations on standard unseen attacks (e.g., AutoAttack, CW-L2, and DeepFool) reported AUROC values around 84–86%, further supporting the robustness of the proposed pipeline under distribution shifts.

#### 6.7.2. Adversarial Transferability Resistance

We further evaluate AEGIS under black-box transfer attacks, where adversarial examples generated on surrogate models are evaluated against the proposed pipeline. In this setting, adversarial samples are crafted using FGSM and PGD on external architectures (ResNet-50 and DenseNet-121), while AEGIS is trained on ResNet-18. This setup simulates realistic scenarios where an attacker does not have access to the target model.

The results in Table 7 demonstrate that AEGIS exhibits strong resilience to transferable adversarial perturbations, with AUROC consistently above 88% across all settings. In addition, entropy values remain elevated under transferred attacks, indicating that the evidential classifier effectively captures increased epistemic uncertainty in black-box scenarios. These findings confirm that the combination of semantic filtering and instability-driven evidential reasoning significantly reduces the effectiveness of transfer-based adversarial attacks.

#### 6.7.3. Threat Model Resolution Mapping

To evaluate the security coverage of AEGIS, we map each threat category to its corresponding empirical performance.

- $l_{p}$-bounded gradient-based attacks (FGSM, PGD): These attacks are effectively mitigated through augmentation-driven instability profiling and evidential uncertainty estimation. High AUROC values for FGSM (96.1%) and PGD (94.2%) reported in Table 2 confirm strong separability between clean and adversarial samples. Furthermore, Table 5 shows increased entropy and higher calibration error under attack, supporting reliable uncertainty-aware rejection.
- Localized spatial attacks (adversarial patches): SemantiGAN provides effective semantic-level filtering, achieving 93.4% precision and 89.6% recall for adversarial detection (Table 4). This corresponds to a measurable improvement in downstream classification accuracy (+4.5%), indicating strong mitigation of patch-based perturbations that disrupt localized regions while preserving global semantics.
- Semantic and functional attacks: Functional perturbations are addressed through the combined effect of semantic discrimination and instability-based detection, achieving 89.4% AUROC (Table 2). Elevated entropy values in Table 5 further confirm increased epistemic uncertainty under these transformations.
- Geometric transformations: Geometric attacks constitute the most challenging adversarial category, achieving the lowest AUROC (88.3%) among all evaluated attack types. Nevertheless, this result still demonstrates the robustness of AEGIS against complex spatial perturbations. Unlike gradient-based attacks (e.g., FGSM and PGD), which mainly modify pixel intensities while preserving the spatial structure of the image, geometric attacks manipulate the spatial arrangement of semantic features through transformations such as rotation, translation, scaling, shearing, and perspective distortion. As a result, the instability representations extracted by LAFANet become less discriminative because part of the observed feature variation originates from legitimate geometric transformations rather than adversarial manipulations. Furthermore, the semantic-preserving augmentations employed by AEGIS exhibit partial invariance to certain geometric transformations, thereby reducing the separability between clean and adversarial samples.
- Cross-model transfer attacks: As shown in Table 7, AEGIS maintains transfer detection accuracy above 86.5% and AUROC above 88.2%, demonstrating robustness in black-box settings where adversarial examples are generated on surrogate architectures.
- Zero-day adversarial attacks: Evaluation on hybrid PGD+geometric attacks (Table 6) yields an AUROC of 86.5%, confirming generalization to unseen perturbation compositions. The observed confidence drop (−4.2%) relative to known attacks reflects increased difficulty under distribution shift.
- Evidential calibration behavior: EDL-based uncertainty estimation consistently shows increased entropy and higher ECE under adversarial conditions (Table 5), enabling reliable detection of overconfident misclassifications and supporting safe rejection decisions.

### 6.8. Comparison with State-of-the-Art

To evaluate the effectiveness of AEGIS, we conduct a comprehensive comparison against representative state-of-the-art methods. The selected baselines include ODIN [18], which exploits input preprocessing for out-of-distribution detection; LID [26] and Mahalanobis Distance [25], which leverage feature-space geometry; MC Dropout [49], a Bayesian approximation technique for uncertainty estimation; Defense-GAN [39], a generative reconstruction-based defense; DUQ [76], which performs deterministic uncertainty quantification; ALOHA [77], a linearity-aware adversarial detector; Semantic-Aware Adversarial Examples (SAEs) [78]; and the Conflict-Aware Evidential Deep Learning (C-EDL) framework [79]. Table 8 summarizes the results of this empirical evaluation on the Image-Net dataset based on AUROC, AUPRC, Accuracy, ECE, Brier score metrics. As observed, AEGIS consistently achieves the best overall performance across all evaluation metrics, obtaining the highest AUROC (92.1%), AUPRC (90.2%), and classification accuracy (90.7%), while simultaneously yielding the lowest ECE (1.6%) and Brier Score (0.089). The superior performance demonstrates that integrating semantic reasoning, feature instability analysis, and evidential uncertainty modeling produces more discriminative and better-calibrated predictions than existing single-paradigm approaches. The comparative analysis further reveals several limitations of current state-of-the-art methods. Geometry-based approaches, such as LID and Mahalanobis Distance, effectively detect feature-space distribution shifts but do not explicitly capture semantic consistency, limiting their effectiveness against semantic-preserving adversarial manipulations. Bayesian uncertainty estimation methods, represented by MC Dropout, improve predictive uncertainty but often suffer from increased calibration errors and reduced robustness under severe distribution shifts. Reconstruction-based approaches, such as Defense-GAN, enhance robustness by projecting adversarial samples onto the learned data manifold; however, their performance strongly depends on reconstruction quality and incurs substantial computational overhead. Likewise, uncertainty-aware methods, including DUQ and C-EDL, provide calibrated confidence estimates but do not explicitly characterize semantic transformations or feature instability induced by adversarial perturbations. Although an SAE incorporates semantic information into the detection process, it primarily emphasizes semantic consistency and does not jointly exploit instability-aware representations and evidential uncertainty for robust adversarial characterization. In contrast, AEGIS introduces a unified adversarial detection framework that jointly exploits semantic reasoning, feature-space analysis, test-time instability profiling, and evidential uncertainty learning within a single architecture. Rather than relying on a single detection principle, AEGIS constructs an instability feature representation from multiple semantic-preserving augmentations and combines it with Evidential Deep Learning to produce both accurate predictions and well-calibrated uncertainty estimates. Furthermore, unlike the majority of existing adversarial detectors, which formulate the problem as binary adversarial detection, AEGIS performs fine-grained multi-class adversarial attack categorization, enabling simultaneous detection, attack identification, and uncertainty quantification.

### 6.9. Ablation Study

To quantify the contribution of each component in AEGIS, we perform an ablation study by selectively removing key modules, including the SemantiGAN discriminator, stochastic augmentation pipeline, handcrafted instability features, and the Evidential Deep Learning (EDL) classifier. The results are reported in Table 9.

The full AEGIS configuration achieves the best performance across all evaluation metrics, confirming the complementary role of its components. In particular, removing any individual module leads to a consistent degradation in both detection performance and calibration quality, demonstrating that each stage contributes meaningfully to the overall robustness of the system.

### 6.10. Work Limitations

While AEGIS demonstrates strong robustness against a wide range of adversarial attacks, several limitations remain that highlight opportunities for future improvement.

- Geometric robustness: Although AEGIS achieves strong performance against geometric attacks, this category remains the most challenging due to the spatial distortions introduced by some transformations. Unlike additive perturbations, these transformations alter the spatial arrangement of semantic features, making instability representations less discriminative. These observations suggest several promising directions for improving robustness against geometric attacks. This include investigating geometry-aware feature learning through group-equivariant or rotation-equivariant convolutional networks, stronger geometry-aware adversarial training with affine and perspective transformations, and transformation-invariant feature representations or attention-based spatial alignment mechanisms to further enhance robustness against geometric adversarial attacks [80,81].
- Zero-day generalization: AEGIS maintains an AUROC above 86% on zero-day adversarial examples; however, the observed reduction in confidence relative to known attacks (approximately 4%) indicates reduced stability under highly novel perturbation distributions.
- Real-time deployment: All experiments were conducted in an offline setting. As such, latency, throughput, and computational efficiency under real-time constraints have not yet been evaluated, particularly on edge or embedded hardware.
- Physical-world attacks: The current evaluation does not include fully physical adversarial scenarios, such as printed patches, environmental perturbations, or real-world viewpoint distortions. Assessing robustness under such conditions remains an open challenge.
- Scalability to higher resolutions: While most experiments were conducted on Tiny ImageNet (64×64), preliminary results on ImageNet-128 indicate that AEGIS generalizes well to higher resolutions, with only a minor degradation in performance (approximately 2% AUROC drop). This suggests good scalability, although further evaluation on full-resolution datasets is required.

The ablation analysis (Table 9) demonstrates that removing any individual component, SemantiGAN, LAFANet, instability feature construction, or EDL, results in a measurable degradation in detection accuracy, AUROC, and calibration quality. The full AEGIS pipeline achieves the best overall performance, confirming the complementary nature of its components. Specifically:

- SemantiGAN provides early-stage semantic filtering, reducing the propagation of high-risk inputs to downstream modules.
- LAFANet captures instability under stochastic transformations, revealing sensitivity patterns not visible in standard confidence scores.
- Evidential Deep Learning enables calibrated prediction and explicit uncertainty quantification, supporting safe rejection under ambiguity.

Together, these components form a hierarchical defense strategy that integrates semantic reasoning, stability analysis, and uncertainty modeling into a unified adversarial detection framework.

## 7. Conclusions

This work presented AEGIS, a modular adversarial detection framework that integrates semantic filtering via a multi-class GAN discriminator, augmentation-driven instability profiling, and evidential deep learning for calibrated and uncertainty-aware classification. Extensive evaluations on Tiny ImageNet demonstrate that AEGIS consistently achieves high detection performance, strong calibration, and robust resilience against diverse adversarial threat models, including gradient-based, spatial, semantic, and transferable attacks.

The semantic discriminator enables early rejection of semantically inconsistent inputs, reducing the propagation of adversarial signals to downstream components. In parallel, LAFANet captures instability under stochastic transformations, providing discriminative features that reveal adversarial sensitivity beyond raw confidence scores. Finally, the evidential learning module enables principled uncertainty estimation, improving both robustness and interpretability of the final decision process.

While the current evaluation is conducted on controlled benchmarks, including Tiny ImageNet and ImageNet-128, the framework is designed to generalize to more complex real-world scenarios characterized by real time requirements, higher resolution, domain shift, and environment-induced variability. Future work will focus on optimizing low-latency inference for edge devices, extending the framework to privacy-preserving collaborative learning paradigms such as federated learning, and evaluating robustness under fully physical adversarial conditions, including printed and dynamic perturbations. Additional research directions include extending AEGIS to emerging adversarial attack categories and adapting the framework to real-time video analytics and multi-sensor vision systems.

## Figures and Tables

**Table 1:** Dataset partitioning for AEGIS training and evaluation.

Split	Clean	Adversarial (5 Types)	Usage
Train	100 k	100 k (generated)	SemantiGAN
Train	100 k	NA	LAFANet
Train	100 k	NA	EDL
Validation	10 k	10 k	Hyperparameter tuning
Test	10 k	50 k (balanced across 5 attacks)	Final evaluation

**Table 2:** Detection performance of AEGIS across adversarial attack types using AUROC, AUPRC, F1 score, and Accuracy metrics.

Attack Type	AUROC (%)	AUPRC (%)	F1 Score (%)	Accuracy (%)
FGSM	96.1	94.8	94.7	95.3
PGD	94.2	92.5	92.3	93.0
Patch	91.0	88.9	88.5	89.2
Geometric	88.3	86.2	85.1	86.0
Functional	89.4	87.5	86.0	87.1
Average	91.8	89.98	89.32	90.12

**Table 3:** Preliminary AEGIS results on ImageNet-128.

Metric	Tiny ImageNet (64 × 64)	ImageNet-128 (128 × 128)
Accuracy (%)	90.1	88.3
AUROC (%)	91.8	89.7
ECE (%)	1.6	2.2

**Table 4:** Performance of the SemantiGAN discriminator as a semantic filtering module.

Metric	Value (%)	Remarks
Precision (Adversarial)	93.4	High confidence in adversarial detection
Recall (Adversarial)	89.6	Strong detection coverage across attack types
False Rejection Rate (Clean)	3.8	Low misclassification of benign inputs
Classifier Accuracy (w/o Filter)	86.2	Baseline classifier without semantic filtering
Classifier Accuracy (w/ Filter)	90.7	Improved reliability with SemantiGAN
Optimal Threshold	0.72	Selected for best precision–recall trade-off

**Table 5:** Uncertainty -based performance of the Evidential Deep Classifier across clean and adversarial samples.

Input Type	Brier Score ↓	ECE (%) ↓	Entropy ↑
Clean Samples	0.087	1.8	0.23
FGSM	0.122	5.4	1.12
PGD	0.134	6.1	1.45
Patch-based	0.118	5.9	1.33
Geometric	0.129	6.7	1.38
Functional	0.131	6.5	1.41

**Table 6:** Detection performance on zero-day adversarial examples.

Metric	Zero-Day AUROC (%)	ECE (%)	Entropy (↑)
Detection Performance	86.5	4.9	1.48
Confidence Drop (vs. Known Attacks)	−4.2	+2.1	+0.91

**Table 7:** Cross -model adversarial transfer detection performance.

Source Model	Attack Type	Transfer Accuracy (%)	AUROC (%)	Entropy (↑)
ResNet-50	FGSM	89.4	91.2	1.25
ResNet-50	PGD	85.7	89.1	1.38
DenseNet-121	FGSM	88.6	90.3	1.19
DenseNet-121	PGD	84.3	88.2	1.43

**Table 8:** Comparison with state-of-the-art methods.

Method	AUROC (%)	AUPRC (%)	Accuracy (%)	ECE (%)	Brier Score
ODIN [18]	88.7	85.2	86.1	4.2	0.117
LID [26]	89.5	86.7	87.5	3.8	0.112
MC Dropout [49]	86.4	82.8	85.0	5.1	0.124
Defense-GAN [39]	90.2	87.9	88.6	3.4	0.106
Mahalanobis [25]	89.1	86.3	87.2	3.7	0.111
DUQ [76]	89.6	86.8	88.0	3.1	0.104
ALOHA [77]	90.4	88.3	89.0	2.9	0.099
SAE [78]	91.0	88.9	89.4	2.5	0.095
C-EDL [79]	91.6	89.6	90.1	2.0	0.092
AEGIS	92.1	90.2	90.7	1.6	0.089

**Table 9:** Ablation study of AEGIS components on detection and calibration performance.

Configuration	AUROC (%)	AUPRC (%)	Accuracy (%)	ECE (%)	Brier Score
Full AEGIS Pipeline	92.1	90.2	90.7	1.6	0.089
w/o SemantiGAN	87.5	84.6	86.2	3.9	0.104
w/o Stochastic Augmentation	88.1	85.3	87.1	3.6	0.101
w/o Instability Features	88.7	86.1	87.4	3.3	0.096
Softmax instead of EDL	85.8	83.7	85.2	4.4	0.108
No Defense (Baseline)	80.2	78.5	82.3	5.7	0.117

## Data Availability

The data presented in this study are available on request from the corresponding author.

## References

[1] Foukalas F. (2025). A survey of artificial neural network computing systems. Cogn. Comput..

[2] Vadillo J., Santana R., Lozano J.A. (2025). Adversarial attacks in explainable machine learning: A survey of threats against models and humans. Wiley Interdiscip. Rev. Data Min. Knowl. Discov..

[3] Yerlikaya F.A., Bahtiyar Ş. (2026). A survey of adversarial attacks on machine learning. Neurocomputing.

[4] Kurakin A., Goodfellow I., Bengio S. (2017). Adversarial examples in the physical world. Proceedings of the Artificial Intelligence Safety and Security.

[5] Papernot N., McDaniel P., Jha S., Fredrikson M., Celik Z.B., Swami A. (2016). The limitations of deep learning in adversarial settings. Proceedings of the IEEE European Symposium on Security and Privacy (EuroS&P).

[6] Tran N.N., Bui A.T., Phung D., Le T. (2022). Multiple Perturbation Attack: Attack Pixelwise Under Different p norms For Better Adversarial Performance. arXiv.

[7] Villegas-Ch W., Jaramillo-Alcázar A., Luján-Mora S. (2024). Evaluating the robustness of deep learning models against adversarial attacks: An analysis with fgsm, pgd and cw. Big Data Cogn. Comput..

[8] Chen Z., Wang C., Crandall D. (2022). Semantically stealthy adversarial attacks against segmentation models. Proceedings of the IEEE/CVF Winter Conference on Applications of Computer Vision.

[9] Zhang Z., Li X., Zhang P., Kui W., Gao T., Shen T. (2025). AttackTracer: Semantic-level adversarial attack location traceability via evidential diffusion model. Neurocomputing.

[10] Brown T.B., Mané D., Roy A., Abadi M., Gilmer J. (2017). Adversarial patch. arXiv.

[11] Xiao C., Zhu J.Y., Li B., He W., Liu M., Song D. (2018). Spatially transformed adversarial examples. arXiv.

[12] Swain S.K., Kumar V., Kim D.D., Bai G. (2023). SPAT: Semantic-preserving adversarial transformation for perceptually similar adversarial examples. ECAI 2023.

[13] Yin M., Li S., Cai Z., Song C., Asif M.S., Roy-Chowdhury A.K., Krishnamurthy S.V. (2021). Exploiting multi-object relationships for detecting adversarial attacks in complex scenes. Proceedings of the IEEE/CVF International Conference on Computer Vision.

[14] Hosseini H., Poovendran R. (2018). Semantic adversarial examples. Proceedings of the IEEE Conference on Computer Vision and Pattern Recognition Workshops.

[15] Song Y., Zhou Z., Lu Q., Zhang H., Hu Y., Xue L., Hu S., Li M., Zhang L.Y. (2026). Segtrans: Transferable adversarial examples for segmentation models. IEEE Trans. Multimed..

[16] Madry A., Makelov A., Schmidt L., Tsipras D., Vladu A. (2017). Towards deep learning models resistant to adversarial attacks. arXiv.

[17] Feinman R., Curtin R.R., Shintre S., Gardner A.B. (2017). Detecting adversarial samples from artifacts. arXiv.

[18] Liang S., Li Y., Srikant R. (2017). Enhancing the reliability of out-of-distribution image detection in neural networks. arXiv.

[19] Zhou Y., Kantarcioglu M. (2018). Classification by re-generation: Towards classification-based adversarial detection. arXiv.

[20] Liu Z., Li X., Liang H., Snasel V., Ojha V. (2025). AdaGAT: Adaptive Guidance Adversarial Training for the Robustness of Deep Neural Networks. Proceedings of the Chinese Conference on Pattern Recognition and Computer Vision (PRCV).

[21] Ye J., Yu R., Liu S., Wang X. (2024). Mutual-modality adversarial attack with semantic perturbation. Proc. AAAI Conf. Artif. Intell..

[22] Swami C.P., Joshi D. (2025). Investigating the Impact of Adversarial Attacks on Deep Learning-Based Wearable Robot Controllers: Security, Reliability, and Safety Concerns. IEEE Trans. Ind. Inform..

[23] Li J., Wu H., Ren Y., Zhang J., Shen L. (2026). Two-stage uncertainty-aware adversarial patch attack for semantic segmentation. Int. J. Intell. Comput. Cybern..

[24] Carlini N., Wagner D. (2017). Towards evaluating the robustness of neural networks. Proceedings of the 2017 IEEE Symposium on Security and Privacy (SP).

[25] Lee K., Lee K., Lee H., Shin J. (2018). A simple unified framework for detecting out-of-distribution samples and adversarial attacks. Advances in Neural Information Processing Systems.

[26] Ma X., Li B., Wang Y., Erfani S.M., Wijewickrema S., Schoenebeck G., Song D., Houle M.E., Bailey J. (2018). Characterizing adversarial subspaces using local intrinsic dimensionality. arXiv.

[27] Goyal S., Alayrac J.B., Kovacs A., Kohli P. Detection of adversarial examples using robustness discrepancies. Proceedings of the Advances in Neural Information Processing Systems (NeurIPS).

[28] Uvdal P., Mirkhalaf M. (2025). Test-time data augmentation: Improving predictions of recurrent neural network models of composites. Eng. Appl. Artif. Intell..

[29] Li X., Li F. (2017). Adversarial examples detection in deep networks with convolutional filter statistics. Proceedings of the IEEE International Conference on Computer Vision.

[30] Zhou D., Wang N., Liu T., Gao X. (2025). Improving adversarial training from the perspective of class-flipping distribution. IEEE Trans. Pattern Anal. Mach. Intell..

[31] Xiao Y., Chen S., Chen K., Guan Q., Liu Z. (2025). Towards Adversarial Patch Attacks on Deep Crowd-Counting Networks via Density-aware Normalized Feature Learning. Knowl.-Based Syst..

[32] Hasanebrahimi A., Baghbaderani B.K., Hosseini R., Kalhor A. (2023). Density estimation helps adversarial robustness. Proceedings of the 2023 13th International Conference on Computer and Knowledge Engineering (ICCKE).

[33] Guesmi A., Shafique M. (2025). DRIFT: Divergent Response in Filtered Transformations for Robust Adversarial Defense. arXiv.

[34] Jia C., Wu Z., Su C., Liu H., Xiao Y. (2025). Adversarial defense method to face forgery detection based on masked conditional diffusion model. Expert Syst. Appl..

[35] Lao D., Zhang Y., Oskouie H.E., Wu Y., Wong A., Soatto S. (2025). Test-Time Defense Against Adversarial Attacks via Stochastic Resonance of Latent Ensembles. arXiv.

[36] Sai U.D., Yogeesh V.S., Vindya N., Mulgund A.P., Das B. (2024). Interpretation of white box adversarial attacks on machine learning model using grad-CAM. Proceedings of the 2024 8th International Symposium on Innovative Approaches in Smart Technologies (ISAS).

[37] Cao Y., Xing Y., Zhang J., Lin D., Zhang T., Tsang I., Liu Y., Guo Q. (2025). Scenetap: Scene-coherent typographic adversarial planner against vision-language models in real-world environments. Proceedings of the Computer Vision and Pattern Recognition Conference.

[38] Gong Y., Wang S., Jiang X., Yin L., Sun F. (2023). Adversarial example detection using semantic graph matching. Appl. Soft Comput..

[39] Samangouei P., Kabkab M., Chellappa R. Defense-GAN: Protecting classifiers against adversarial attacks using generative models. Proceedings of the International Conference on Learning Representations (ICLR).

[40] Akcay S., Atapour-Abarghouei A., Breckon T.P. (2018). Ganomaly: Semi-supervised anomaly detection via adversarial training. Proceedings of the Asian Conference on Computer Vision.

[41] Xu L., Yang Z., Zhao D., Yu F., Zhou Y., Zhang H. (2025). G-VAE: Variational autoencoder-based adversarial attacks and defenses in industrial control systems. Comput. Electr. Eng..

[42] Qian C., Tang W., Wang Y. (2025). RGAnomaly: Data reconstruction-based generative adversarial networks for multivariate time series anomaly detection in the Internet of Things. Future Gener. Comput. Syst..

[43] Xiao C., Li B., Zhu J.Y., He W., Liu M., Song D. (2018). Generating adversarial examples with adversarial networks. arXiv.

[44] Jiang Z., Chen T., Chen T., Wang Z. (2020). Robust pre-training by adversarial contrastive learning. Advances in Neural Information Processing Systems.

[45] He Z., Chen Y., Chen A., Zhang D., Zhang H., Zhang J. (2025). GAN-enabled U-shaped network for adversarial attack generation for autonomous unmanned vehicles. IEEE Trans. Autom. Sci. Eng..

[46] Ge J.W., Cao J.X., Zhao Z.X., Liu B. (2025). Fsd-gan: Generative adversarial training for face swap detection via the latent noise fingerprint. J. Comput. Sci. Technol..

[47] Fakour F., Mosleh A., Ramezani R. (2024). A structured review of literature on uncertainty in machine learning & deep learning. arXiv.

[48] Tuna O.F., Catak F.O., Eskil M.T. (2022). Exploiting epistemic uncertainty of the deep learning models to generate adversarial samples. Multimed. Tools Appl..

[49] Gal Y., Ghahramani Z. (2016). Dropout as a bayesian approximation: Representing model uncertainty in deep learning. Proceedings of the International Conference on Machine Learning.

[50] Lakshminarayanan B., Pritzel A., Blundell C. (2017). Simple and scalable predictive uncertainty estimation using deep ensembles. Advances in Neural Information Processing Systems.

[51] Sensoy M., Kaplan L., Kandemir M. (2018). Evidential deep learning to quantify classification uncertainty. Advances in Neural Information Processing Systems.

[52] Wang Y., Sun T., Li S., Yuan X., Ni W., Hossain E., Poor H.V. (2023). Adversarial attacks and defenses in machine learning-empowered communication systems and networks: A contemporary survey. IEEE Commun. Surv. Tutor..

[53] He Y., Bi Y., Li L., Pun C.M., Jiao W., Jin Z. (2024). Mutual evidential deep learning for semi-supervised medical image segmentation. Proceedings of the 2024 IEEE International Conference on Bioinformatics and Biomedicine (BIBM).

[54] Holmquist K., Klasén L., Felsberg M. (2023). Evidential deep learning for class-incremental semantic segmentation. Proceedings of the Scandinavian Conference on Image Analysis.

[55] Szegedy C., Zaremba W., Sutskever I., Bruna J., Erhan D., Goodfellow I., Fergus R. Intriguing properties of neural networks. Proceedings of the International Conference on Learning Representations (ICLR).

[56] Goodfellow I.J., Shlens J., Szegedy C. (2015). Explaining and harnessing adversarial examples. arXiv.

[57] Liu H., Ge Z., Zhou Z., Shang F., Liu Y., Jiao L. (2023). Gradient correction for white-box adversarial attacks. IEEE Trans. Neural Netw. Learn. Syst..

[58] Zhu Y., Zhao Y., Hu Z., Luo T., He L. (2024). A review of black-box adversarial attacks on image classification. Neurocomputing.

[59] Costa J.C., Roxo T., Proença H., Inácio P.R. (2026). LISArD: Learning image similarity to defend against gray-box adversarial attacks. PeerJ Comput. Sci..

[60] Ali U.A., Dogra K., Sharma S. (2025). White-box adversarial exploitation of NIDS: Insights from FGSM, PGD, and C&W. Proceedings of the 2025 2nd International Conference on Computational Intelligence, Communication Technology and Networking (CICTN).

[61] Rub R., Noor S., Usmani I.A., Ali Z.A. (2026). PGD–PPM: A Hybrid Framework for Enhancing Adversarial Robustness in Traffic Sign Recognition System. IEEE Access.

[62] Lian J., Mei S., Zhang S., Ma M. (2022). Benchmarking adversarial patch against aerial detection. IEEE Trans. Geosci. Remote Sens..

[63] Joshi A., Mukherjee A., Sarkar S., Hegde C. (2019). Semantic adversarial attacks: Parametric transformations that fool deep classifiers. Proceedings of the IEEE/CVF International Conference on Computer Vision.

[64] Chen G., Zhang Z., Peng Y., Li C., Li T. (2025). G&G Attack: General and Geometry-Aware Adversarial Attack on the Point Cloud. Appl. Sci..

[65] Papernot N., McDaniel P., Swami A., Harang R. (2016). Transferability in machine learning: From phenomena to black-box attacks using adversarial samples. arXiv.

[66] Hein M., Andriushchenko M. (2019). Why ReLU networks yield high-confidence predictions far away from the training data and how to mitigate the problem. Proceedings of the IEEE Conference on Computer Vision and Pattern Recognition (CVPR).

[67] Girardi P., Vesely A., Lakens D., Altoè G., Pastore M., Calcagnì A., Finos L. (2024). Post-selection inference in multiverse analysis (PIMA): An inferential framework based on the sign flipping score test. Psychometrika.

[68] Garg S., Erickson N., Sharpnack J., Smola A., Balakrishnan S., Lipton Z.C. (2023). Rlsbench: Domain adaptation under relaxed label shift. Proceedings of the International Conference on Machine Learning.

[69] Xu K., Zhang L., Shi J. (2025). Good seed makes a good crop: Discovering secret seeds in text-to-image diffusion models. Proceedings of the 2025 IEEE/CVF Winter Conference on Applications of Computer Vision (WACV).

[70] Esposito M., Valente G., Plasencia-Calaña Y., Dumontier M., Giordano B.L., Formisano E. (2024). Bridging auditory perception and natural language processing with semantically informed deep neural networks. Sci. Rep..

[71] Ye Y., Li Y., Ouyang R., Zhang Z., Tang Y., Bai S. (2023). Improving machine learning based phase and hardness prediction of high-entropy alloys by using Gaussian noise augmented data. Comput. Mater. Sci..

[72] Zhang H., Zhao B., Ma X., Han L., Yang M., Ulfarsson M.O., Sigurdsson J., Benediktsson J.A. (2026). Hyperspectral Image Mixed Noise Removal Based on Sparsity Constraint and Low-Rank Approximation. IEEE J. Sel. Top. Appl. Earth Obs. Remote Sens..

[73] Zhang R., Ng M.K., Ljubenovic M., Zhuang L. (2026). Unsupervised Hyperspectral Image Denoising via Spectral Learning Preference of Neural Networks. Remote Sens..

[74] Shao M., Tan X., Shang K., Liu T., Cao X. (2025). A Hybrid Model of State Space Model and Attention for Hyperspectral Image Denoising. IEEE J. Sel. Top. Appl. Earth Obs. Remote Sens..

[75] Emde C., Pinto F., Lukasiewicz T., Torr P., Bibi A. (2025). Towards certification of uncertainty calibration under adversarial attacks. Proceedings of the International Conference on Learning Representations.

[76] Van Amersfoort J., Smith L., Teh Y.W., Gal Y. (2020). Uncertainty estimation using a single deep deterministic neural network. Proceedings of the International Conference on Machine Learning (ICML).

[77] Carlini N., Athalye A., Papernot N., Brendel W., Rauber J., Tsipras D., Goodfellow I., Madry A., Kurakin A., Tramèr F. (2022). Detecting adversarial examples from artifacts. Proceedings of the IEEE Symposium on Security and Privacy (S&P).

[78] Zhang A., Zhang M., Wischik D. (2024). Constructing semantics-aware adversarial examples with a probabilistic perspective. Advances in Neural Information Processing Systems.

[79] Barker C., Bethell D., Gerasimou S. Robust Adversarial Quantification Via Conflict-Aware Evidential Deep Learning. Proceedings of the Fourteenth International Conference on Learning Representations.

[80] Wang L., Uddin I.I., Zhang C., Qin X., Zhou Y., Zhang J., Liu Z., Lu S., Liu X. (2026). Bridging symmetry and robustness: On the role of equivariance in enhancing adversarial robustness. Advances in Neural Information Processing Systems.

[81] Deng Z., Zhao Q., Ye L., He Q., He Z., Huang J. (2025). A geometry-aware generative model for face morphing attacks. Knowl.-Based Syst..

